# Expanded Dengue Syndrome Presenting as Intracranial Hemorrhage, Fever, and Rhabdomyolysis: A Case Report

**DOI:** 10.7759/cureus.34252

**Published:** 2023-01-26

**Authors:** Shubhajeet Roy, Shikhar S Gupta, Shashank Prajapati, Syed N Muzaffar, Avinash Agrawal

**Affiliations:** 1 Department of Medicine, King George's Medical University, Lucknow, IND; 2 Department of Medical Sciences, King George's Medical University, Lucknow, IND; 3 Department of Critical Care Medicine, King George's Medical University, Lucknow, IND

**Keywords:** myoglobinuria, rhabdomyolysis, intracranial hemorrhages, severe dengue, dengue

## Abstract

Rare clinical manifestations of dengue are included under the expanded dengue syndrome (EDS), with intracranial hemorrhage (ICH) being one of them. We discuss an uncommon presentation of dengue with basal ganglia hemorrhage, hyperthermia, and rhabdomyolysis in a 53-year-old hypertensive female who presented with sudden onset syncope, left-sided weakness, and altered sensorium for days, with high-grade fever and vomiting. The Glasgow coma scale (GCS) score was 5, and the patient was intubated. Noncontrast computerized tomography (NCCT) of the brain revealed right basal ganglia bleeding with intraventricular hemorrhage. Electrocardiography (ECG) revealed sinus tachycardia. The patient had spikes of high-grade fever, rhabdomyolysis, stage III acute kidney disease, and coagulopathy. Dengue IgM antibodies were positive. Treatment was started, and the patient was in the intensive care unit (ICU) for six months, following which she was discharged. Given this, one can speculate on the importance of viral diseases presenting with ICH as these are rare and are diagnosed quite late, which can also prove to be fatal.

## Introduction

Dengue virus, a member of the Flaviviridae family, has a single-stranded RNA genome. Dengue is currently endemic in many parts of the world and is transmitted primarily through the bite of the Aedes aegypti mosquito. It has mainly four closely related serotypes: dengue virus-1 (DENV-1), DENV-2, DENV-3, and DENV-4. It can manifest in three forms: dengue fever (DF), dengue hemorrhagic fever (DHF), and dengue shock syndrome (DSS) [1]. Frequently, some manifestations of dengue do not fall into either of the categories and are grouped under expanded dengue syndrome (EDS), with intracranial hemorrhage (ICH) being one of them. The global dengue burden has risen in the past few years, but the case reporting during 2020 and 2021 was low, attributed to the global COVID-19 pandemic [1].

## Case presentation

**Figure 1:**
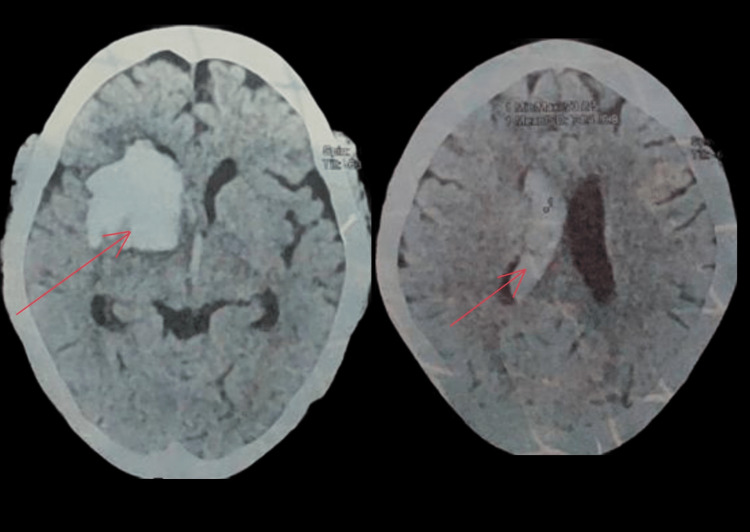
NCCT of the head showing right basal ganglia bleeding with interventricular hemorrhage. NCCT, noncontrast computerized tomography

A 53-year-old morbidly obese female with a history of hypertension presented to the emergency department (ED), with a sudden onset of loss of consciousness, left-sided weakness, and altered sensorium for two days, which was associated with high-grade fever and vomiting. At admission, she was unconscious (Glasgow coma scale, or GCS, score = 5); had tachypnea (respiratory rate, or RR, 30-35 minute^-1^), tachycardia (heart rate, or HR, 114 bpm), and blood pressure (BP) 153/90 mmHg; was maintaining oxygen saturation of 94% to 94% (on room air) on spontaneous respiration; and was passing urine output around 40-50 mL/hour. Given airway protection, tracheal intubation was performed immediately. Post intubation, the GCS was 6. Noncontrast computerized tomography (NCCT) of the brain revealed right basal ganglia bleeding with intraventricular hemorrhage (Figure 1).

Then she was transferred to ICU, where cerebral protection strategy (head-up position, neck in a neutral position, osmotic diuretics, mean arterial pressure (MAP) 85-90 mm Hg, normoxia, normocarbia, euglycemia, normothermia, and maintenance of sodium (Na+) levels between 145 and 155 mEq/L) and further supportive care was initiated. Laboratory evaluation at admission is summarized in Table 1. Electrocardiography (ECG) showed sinus tachycardia. Chest X-ray (CXR) was normal.

**Table 1:** Patient’s laboratory findings upon admission.

Laboratory parameters	Patient findings	Normal range
Hemoglobin	11.6 g/dL	12-16 g/dL
Total leukocyte count	4,944 cells/mm^3^	4,000-11,000 cells/mm^3^
Platelet count	1.42 lakh cells/mm^3^	1.5-4 lakh cells/mm^3^
Total bilirubin	0.31 mg/dL	0-1 mg/dL
Direct bilirubin	0.19 mg/dL	0-0.2 mg/dL
Aspartate amino transferase	89 IU/L	0-40 IU/L
Alanine amino transferase	75 IU/L	0-48 IU/L
Alkaline phosphatase	464 IU/L	30-115 IU/L
Blood urea nitrogen	121 mg/dL	6-24 mg/dL
Serum creatinine	3.41 mg/dL	0.74-1.35 mg/dL
Serum sodium (Na+)	144 mEq/L	135-145 mEq/L
Serum potassium (K+)	3.13 mEq/L	3.6-5.2 mEq/L

After a few days of ICU admission, she started spiking high-grade temperature (core temperature as high as 106-107 °F), which was associated with rhabdomyolysis (serum creatine kinase (CK-total) = 10,000 IU/L), stage III acute kidney injury (AKI, requiring five sessions of renal replacement therapy), and coagulopathy (requiring blood component therapy). On workup, cultures turned out to be sterile. Other possibilities of hyperthermia (such as drug toxicity) were ruled out. NCCT head was repeated, which did not reveal any hematoma expansion. Workup for COVID-19 was also negative. On account of a history of fever with chill and myalgia around a week before ED admission, a tropical workup was sent for malaria, typhoid, leptospirosis, dengue, chikungunya, and scrub typhus, in which serology (IgM antibodies) for dengue turned out to be positive. Gradually, the temperature trends, renal functions, and coagulopathy recovered. In intercurrent issues, she had septic shock (secondary to ventilator-associated pneumonia and grade IV pressure injury in the sacral region) and neuromuscular weakness, resulting in prolonged weaning. Eventually, the patient was discharged after six months of ICU stay in tracheostomized condition, with a GCS score of 9, accepting Ryle's tube feeding and passing adequate urine output.

## Discussion

The presence of DENV and anti-DENV IgM antibodies in the cerebrospinal fluid (CSF) of dengue patients indicates direct cerebral invasion, which may happen through infected macrophages in the blood-brain barrier [2]. Neurological manifestations of dengue come under the EDS. These neurological manifestations are divided into three categories: (1) related to neurotropic manifestations, such as encephalitis, meningitis, myositis, and myelitis; (2) due to systemic complications, such as encephalopathy, stroke, and hypokalemic paralysis; and (3) postinfection complications, such as encephalomyelitis, optic neuritis, and Guillain-Barre syndrome (GBS) [3]. Hemorrhage in dengue has a multifactorial mechanism secondary to thrombocytopenia, disseminated intravascular coagulopathy (DIC), and hepatic dysfunction [4]. As per available literature, the most common hemorrhages reported in dengue include petechiae, ecchymoses, gastrointestinal bleeds, and epistaxis [5].

ICH is a rare yet challenging issue faced by patients with dengue. Chang et al. conducted a retrospective study of 182 Taiwanese dengue patients during the 2014-2015 outbreak. There were 13 (7.14%) cases of hemorrhage (six of subdural hemorrhage, three of subarachnoid hemorrhage, one of both subdural hemorrhage and subarachnoid hemorrhage, and three of intracerebral hemorrhage) and 26 cases of infarction. The overall mortality of the ICH group was a striking high of 30.8% as opposed to that of 11.5% in the infarction group. Bone pain, arthralgia, dizziness, and altered consciousness were far more prevalent in the ICH and infarction groups as compared to others. The significant independent factor for ICH or infarction was altered consciousness [6]. The largest number of ICH cases (18, 1.1%) reported in a study is, however, from India in 2015, due to DENV-2 [7].

Basal ganglia hemorrhage is a far rare manifestation of dengue. In a 2014 study, three out of 21 serologically confirmed patients with dengue plus altered sensorium had thalamic and basal ganglia hemorrhages [8]. In another 2003-2005 study, out of the 11 patients with febrile encephalopathy, only one had T2 hyperintensity in the globus pallidus on cranial MRI. The patient had presented with prolonged status epilepticus [2].

Our patient also had AKI with rhabdomyolysis. The literature also reported cases of rhabdomyolysis in dengue presenting with myalgia, dark urine, and elevated CK levels without AKI. Other than rhabdomyolysis, hemodynamic instability, hemolysis, glomerular injury, and direct action of viral particles on renal tissue may act as risk factors for the development of AKI [9].

## Conclusions

It may be essential to rule out viral infections such as dengue also in patients where the underlying cause of intracranial hemorrhage is not clear or where the site of intracranial hemorrhage is atypical, especially in patients presenting with acute febrile illness. Although hypertensive bleeding is more common in basal ganglia, still there may be a coexisting viral infection, as was seen in our case. As cases of intracranial hemorrhage are rare and may happen at atypical sites, often these cases are diagnosed late, which may be fatal.
